# First-principles predictions of two-dimensional Ce-based ferromagnetic semiconductors: CeF_2_ and CeFCl monolayers†

**DOI:** 10.1039/d4ra06728b

**Published:** 2025-01-23

**Authors:** Y. Hu, Y. L. Song, Y. H. Huang, S. Y. Cao, Y. Yang

**Affiliations:** a School of Electronic Information, Huzhou College Huzhou 313000 China huyan@zjhzu.edu.cn; b Huzhou Key Laboratory for Urban Multidimensional Perception and Intelligent Computing, Huzhou College Huzhou 313000 China; c College of Physics and Electronic Engineering, Nanyang Normal University Nanyang 473061 China; d School of Physics & Information Technology, Shaanxi Normal University Xi'an 710119 Shaanxi China; e School of Physics and Electronic Information, Yan'an University Yan'an 716000 China

## Abstract

Two-dimensional (2D) ferromagnetic (FM) semiconductors hold great promise for the next generation spintronics devices. By performing density functional theory first-principles calculations, both CeF_2_ and CeFCl monolayers are studied, our calculation results show that CeF_2_ is a FM semiconductor with sizable magneto-crystalline anisotropy energy (MAE) and high Curie temperature (290 K), but a smaller band gap and thermal instability indicate that it is not applicable at higher temperature. Its isoelectronic analogue, the CeFCl monolayer, is a bipolar FM semiconductor, its dynamics, elastic, and thermal stability are confirmed, our results demonstrate promising applications of the CeFCl monolayer for next-generation spintronic devices owing to its high Curie temperature (200 K), stable semiconducting features, and stability. Under biaxial strain from −5% to 5%, the CeFCl monolayer is a semiconductor with sizable MAE, its Curie temperature can increase to 240 K, the easy magnetization axes for CeFCl monolayer are still along the out-of-plane directions because the couplings between Cef_*y*(3*x*^2^–*y*^2^)_ and f_*x*(*x*^2^–3*y*^2^)_ orbitals in the different spin channels contribute most to the MAE according to second-order perturbation theory.

## Abstract 2

The CeFCl monolayer is a ferromagnetic bipolar semiconductor with stable semiconducting features and high Curie temperature.

## Graphical abstract

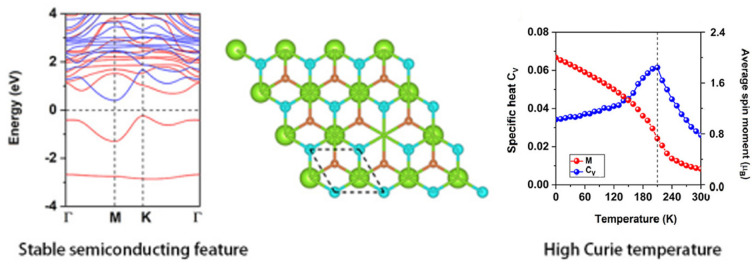

## Introductions

Two-dimensional (2D) ferromagnetic (FM) semiconductors have attracted widespread attention because they hold significant promise for miniaturization of spintronic devices such as quantum computation, high-frequency devices, high-density information storage and so on.^1–3^ Although many efforts were devoted to inducing magnetism into routine semiconductors to design novel 2D FM semiconductors,^4,5^ controlling the distribution of magnetic moments precisely remains a challenging and complicated goal.^6^ In 2017, the first two 2D intrinsic FM semiconductors (monolayer CrI_3_ (ref. 7) and bilayer Cr_2_Ge_2_Te_6_ (ref. 8)) were reported, opening the door for scalable applications related to FM semiconductors.

However, lower Curie temperatures *T*_C_ for both monolayer CrI_3_ (ref. 7) (45 K) and bilayer Cr_2_Ge_2_Te_6_ (ref. 8) (28 K) greatly restricted their application potential. “Is it possible to create magnetic semiconductors that work at room temperature?” is still one of the most challenging 125 big questions in science in this century.^9^ Monolayers including CrX_3_ (X = F, Cl, Br, I),^10^ CrSX (X = Cl, Br, I),^11,12^ MnO_2_,^13^ TiInTe_3_,^14^ h-CrC,^15^ NiCl_2_O_8_,^16^ BiXO_3_ (X = Ru, Os),^17^ RuXY (X = Br, Cl; Y = F, Cl)^18^ and so on^19–31^ were demonstrated to be FM semiconductors, their predicted Curie temperatures range from 23 K to 855 K,^10–31^ for these transition metal compounds, ferromagnetism is induced by super-exchange or double-exchange interaction mediated by delocalized p electrons from sp ions. Previous studies have shown that various methods like strain,^11,15,17^ carrier doping,^30^ and atomic substitution^10–12,17,18^ can all enhance FM interactions between transition metal ions on the 2D FM semiconductors effectively, for example, the *T*_C_ for CrSBr monolayer increases from 80 K to 189 K under the uniaxial strain ranging from −5% to 5%;^11^ by isoelectronic substitution, the *T*_C_ for both RuBrF (367 K) and RuClF (316 K) monolayers are higher than that of the RuBr_2_ monolayer (201 K).^18^

Since monolayers of EuSi_2_ and GdSi_2_ have been synthesized,^32^ the variety of rare-earth (RE) metal monolayers have gradually been enriched both experimentally^33^ and theoretically,^34–46^ their novel physical and chemical properties such as ferro-elasticity,^35^ topological properties,^36^ and valley polarization^37,38^ are also being studied. These monolayers have larger magneto-crystalline anisotropy energies (MAE) which are of benefit for high-density information storage because of heavier RE metal ions.^34–46^ Particularly, among them, GdI_2_,^34^ GdS_2_,^35^ GdSe_2_,^35^ GdIBr^37^ and GdX_2_ (X = Cl, Br, I)^44^ are predicted to be FM semiconductors with high *T*_C_ ranging from 224 K to 648 K, moreover, *T*_C_ for the GdIBr monolayer can increase from 140 K to 245 K upon carrier doping,^37^ indicating FM interactions mediated by d electrons from RE metal ions on the 2D FM semiconductors are considerable and can also be enhanced effectively, but besides Gd-based monolayers, are there any RE metal semiconducting monolayers with high *T*_C_?

In this work, by performing a comprehensive computational study based on the first-principles method, we investigated the stability, electronic and magnetic properties of RE metal monolayer CeF_2_, we find that CeF_2_ monolayer is a kind of novel FM semiconductor with Curie temperature close to room temperature (290 K) and sizable MAE, but smaller band gap indicate larger possibility of thermally induced electronic hopping. By breaking symmetry, the isoelectronic analogues, CeFCl monolayer which may be fabricated by well controlling on the stoichiometric ratio *via* the chemical vapor deposition (CVD) method^47^ were also studied. It has been shown that asymmetric structures may generate bipolar semiconductors which valence band maximum (VBM) and conduction band minimum (VBM) are attributed to different spin channels,^30,35^ comparing with traditional semiconductors, under controlling, 100% spin polarized carriers with reversible polarization directions are more likely to produce for bipolar semiconductors. Moreover, during the electronic hopping process, their spin directions are still unchanged, thus possibility for the thermally induce electronic hopping is always smaller according to the Fermi–Dirac distribution,^48^ rendering semiconductor characteristic is more stable for bipolar semiconductors. Our results show that the CeFCl monolayer is a kind of FM bipolar semiconductor with high Curie temperature, its electronic and magnetic properties under biaxial strain are also been explored.

## Computational methods

In this paper, all the calculations were performed by using the Vienna *ab initio* simulation package (VASP)^49^ based on density functional theory (DFT) method. Electron-ions interactions were described by the projector augmented wave (PAW) method,^50,51^ and the electronic exchange-correlation interactions were described by the Perdew–Burke–Ernzerhof (PBE) functional within the generalized gradient approximation (GGA)^52^ method. The more advanced Heyd–Scuseria–Ernzerh (HSE06) method^53,54^ was adopted for electronic structures calculations, including density of states, band structures and vacuum level, the magneto-crystalline anisotropy energy (MAE) is calculated by taking the spin–orbit coupling (SOC) into account at the PBE level. The Brillouin zone sampling was performed with Monkhorst–Pack *k*-point mesh^55^ of 9 × 9 × 1. The density function perturbation theory (DFPT) method was used to calculate phonon spectrums, phonon calculations were carried out using the Phonopy code^56^ with a 6 × 6 × 1 supercell approach, and the crystal occupation Hamiltonian population (COHP) method employed in LOBSTER was applied to investigate the bonding and anti-bonding states.^57^ An adequate vacuum space of 20 Å was applied along the *z* direction to avoid interactions between periodic layers. *Ab initio* molecular dynamics (AIMD) simulations last for 5 ps with a time step of 2 fs in the NVT ensemble with a temperature fixed at 300 K by the Nose–Hoover method.^58^ The cutoff energy for the plane wave basis set was set as 500 eV. The convergence criterion for the total energy and force were set as 1 × 10^−6^ eV and 0.01 eV Å^−1^, respectively.

## Results and discussions

### Structural properties, ground state and stability of CeF_2_ monolayer

**Fig. 1:**
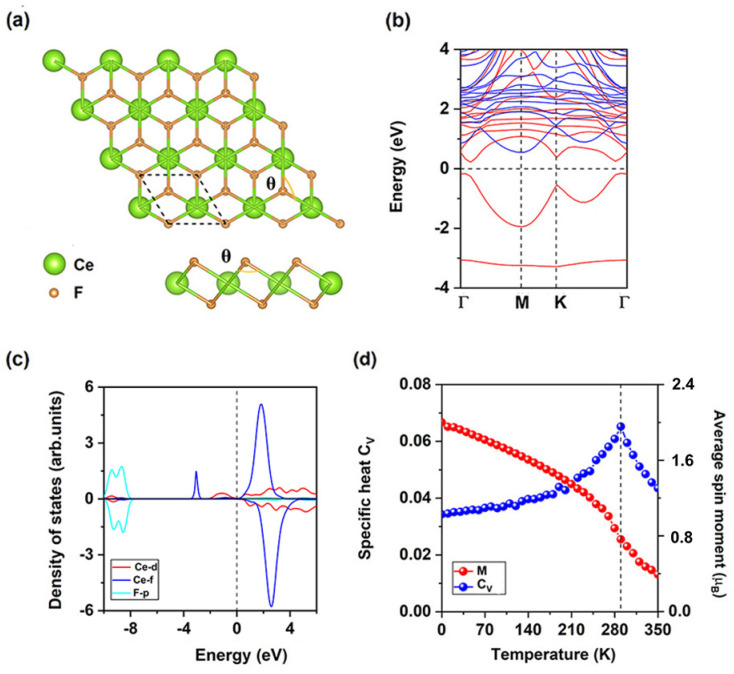
(a) Top and side views for the atomic structures, (b) spin-resolved electronic band structure, (c) projected density of states (PDOS) of Ce-d, Ce-f, and F-p orbitals, (d) the simulated averaged net magnetic moment of CeF_2_ monolayer per formula unit and specific heat with respect to temperature for pristine CeF_2_ monolayer.

The CeF_2_ monolayer belongs to the space group of *P*3̄*m*1, like other 1T monolayer MX_2_ (M: early transition-metal; X: VIIA element), CeF_2_ monolayer has one Ce ionic layer sandwiched by two F ionic layers. As shown in Fig. 1(a), each Ce ion has 6 nearest F ions while each F ion has 3 nearest Ce ions, central Ce ion is settled in a trigonal prismatic configuration. We firstly considered two possible magnetic configurations, namely, ferromagnetic (FM) and antiferromagnetic (AFM) as shown in Fig. S1† to identify its ground state. The calculation results show that CeF_2_ monolayer is ferromagnetic, the energy of the FM state is lower than that of AFM state by 86.45 meV per formula unit. Under its ground state, the optimized lattice constant of CeF_2_ monolayer is 3.65 Å and the nearest Ce–F bond is 2.48 Å, which are both larger than those of monolayer GdF_2_ (3.46 Å and 2.39 Å)^44^ because of larger Ce atomic radius. Moreover, the angle *θ* between nearest Ce ions and F ion is 94.9°.

**Fig. 2:**
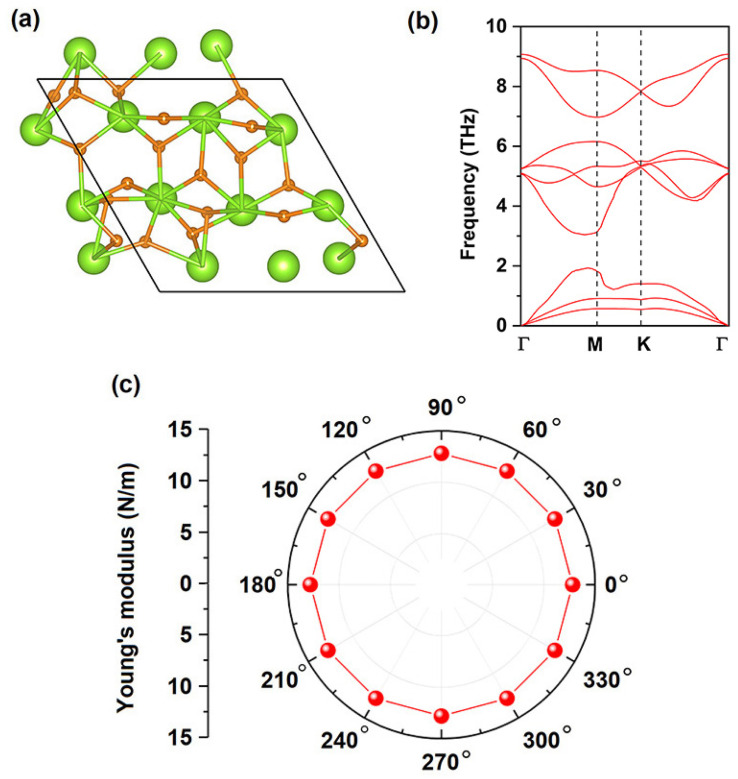
(a) Snapshots of atomic configurations at the end of 5 ps AIMD simulation at 300 K, (b) phonon spectrum, (c) angular dependence of the Young's modulus for CeF_2_ monolayer.

Next, we investigated the stability of CeF_2_ monolayer comprehensively. The formation energy was calculated as:*E*_form_ = *E*_CeF_2__ − *E*_Ce_ − 2*E*_F_where *E*_CeF_2__ is the total energy for CeF_2_ monolayer, *E*_Ce_ and *E*_F_ represent the atomic energies for bulk FCC (face center cubic) Ce and F_2_ gas, the formation energy is −11.97 eV per formula unit for CeF_2_ monolayer, negative value indicates the formation process is exothermic. The AIMD simulation shows that bonds of CeF_2_ monolayer were both broken and reconstruction at 300 K as shown in Fig. 2(a). Moreover, in the whole Brillouin zone, all the phonon branches of CeF_2_ monolayer have real frequencies, confirming its dynamics stability (Fig. 2(b)). Finally, the calculated independent elastic constants *C*_11_ and *C*_12_ are 15.19 N m^−1^ and 6.03 N m^−1^ for CeF_2_ monolayer, respectively, according to the isotropy lattice symmetry,^59^ elastic constant *C*_22_ is equal to *C*_11_, and 
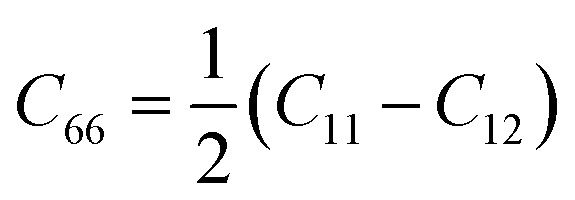
, thus *C*_22_ and *C*_66_ are 15.19 N m^−1^ and 4.58 N m^−1^ respectively. These elastic constants comply with the Born–Huang criteria: *C*_11_ > 0, *C*_11_*C*_22_ − *C*_12_^2^ > 0, and *C*_66_ > 0,^60^ proving the mechanically stability of CeF_2_ monolayer. The orientation-dependent Young's modulus *Y*(*θ*) were calculated *via* the following formula:^61^
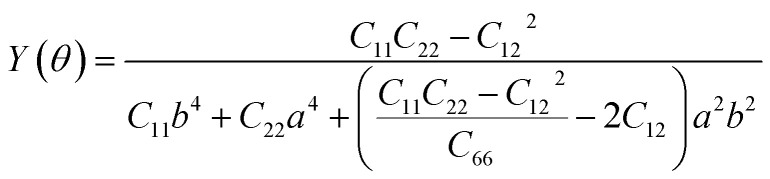
where *a* = cos *θ* and *b* = sin *θ*. As shown in Fig. 2(c), the Young's modulus for CeF_2_ monolayer is isotropy, the value is 12.79 N m^−1^. The gravity-induced out-of-plane deformation h can be evaluated *via* the Young's modulus as:^62^
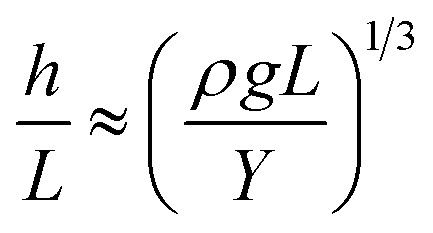
for CeF_2_ monolayer, where the mass density *ρ* is 2.52 × 10^−6^ kg m^−2^, *L* is the size of the monolayer, the gravity acceleration *g* is 9.8 m s^−2^. Taking *L* ≈ 100 μm, we obtain *h*/*L* ≈ 7.63 × 10^−4^, this magnitude is the same as that of synthesized monolayer graphene,^62^ confirming induced deformation is neglectable.

### Electronic and magnetic properties of CeF_2_ monolayer

As shown in Fig. 1(b), CeF_2_ monolayer is an indirect semiconductor with a band gap of 0.40 eV, the valence band maximum (VBM) and conduction band minimum (CBM) are both located in the spin-up channel, they are −0.18 eV and 0.22 eV, respectively. Smaller value of the VBM implying the considerable possibility for the thermally induce electronic hopping, according to the Fermi–Dirac distribution:^48^
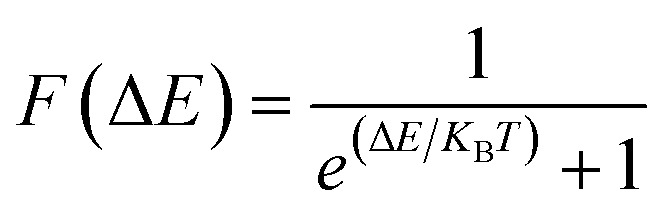
where *F*(Δ*E*) represents the thermally induced occupation probability for valence band electrons, Δ*E* is the energy difference between valence band and conduction band, *K*_B_ is the Boltzmann constant, and *T* is the temperature, at 300 K, this value sharply increases in the order of 10^−7^.

The total magnetic moment is 2 *μ*_B_ per formula unit for CeF_2_ monolayer, magnetic moments for F ions, Ce ions and interstitial are 0.01 *μ*_B_, 1.51 *μ*_B_ and 0.47 *μ*_B_, respectively. Each Ce ion has the same magnetic moment thus indirect exchange interaction between two nearest Ce ions *via* F ions is the super-exchange interaction. As shown in Fig. 1(c), occupied Ce-d and Ce-f electrons align parallelly, since the occupied Ce-f orbitals are highly localized and far away from the Fermi level, Ce-f orbitals are difficult to involved in magnetic coupling, the occupied Ce-d orbitals is the main reason to induce FM super-exchange interaction. According to the GKA (Goodenough–Kanamori–Anderson) rules,^63^ when the angle between two magnetic ions is around 90°, d orbitals of magnetic ions tend to interact with orthometric p orbitals of sp-ions as shown in Fig. S2,† the angle *θ* between nearest Ce ions and F ion is 94.9°, in this way d-electrons from nearest neighboring (NN) Ce ions are FM coupling. Similar to monolayers GdI_2_ (ref. 34–46) and EuSn_2_X_2_,^45^ the Ce-d orbitals are hybridization with F-p orbitals near the Fermi level, using spin-polarized Ce-d electrons as a medium, coupling of f-electrons from NN Ce ions are also FM.

FM super-exchange interaction *via* F ions competes over the AFM direct interaction between two nearest Ce ions, thus CeF_2_ monolayer is FM. The magneto-crystalline anisotropy energy (MAE) is defined as the differences between *E*_*z*_ and *E*_*x*(*y*)_, where *E*_*z*_ and *E*_*x*(*y*)_ are the energies corresponding to magnetization along *z* and *x* (*y*) directions, respectively, the MAE is −2.90 meV per formula unit for CeF_2_ monolayer, the negative value means the easy magnetization axes are along the out-of-plane directions, spin–orbit coupling of Ce-f electrons (−2.88 meV) contribute most to the total MAE. For 2D FM transition metal semiconductors mentioned above,^10–31^ only monolayers BiOsO_3_ (ref. 17) (−5.79 meV) and CrWI_6_ (ref. 31) (5.40 meV) have larger MAE than that of CeF_2_ monolayer.

Moreover, we considered magnetic exchange parameters *J* to describe interactions between the NN magnetic ions, the spin-Hamiltonian is described as:^64^
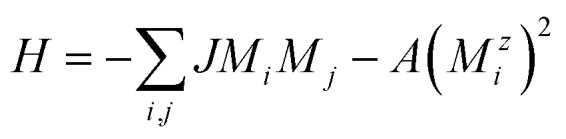
where *J* is the NN magnetic exchange parameter and *A* is the magnetic anisotropy parameter, net, here we choose 2 *μ*_B_, *i* and *j* stand for the NN pair of Ce ions, *M*^*z*^_*i*_ represent net magnetic moment per formula unit along a preferred axis and *A*(*M*^*z*^_*i*_)^2^ is the total MAE. *J* is calculated *via* the energy difference between the FM and AFM states in the 2 × 1 × 1 supercell as:
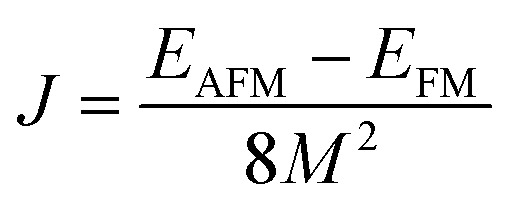


The calculated *J* of NN Ce ions is 5.40 meV, positive/negative value of *J* indicates the preferring of FM/AFM coupling, moreover, calculated *A* is 0.73 meV, *A* is still positive which indicate MAE is benefit for stabilizing long-range magnetic order. The 100 × 100 × 1 supercell containing 20 000 magnetic moment vectors was adopted to perform the Monte Carlo (MC) simulations which lasted for 10^5^ steps at each temperature based on Heisenberg model. Each magnetic moment vector is rotated randomly in two directions of parallel and anti-parallel to the *z* direction. Fig. 1(d) show the evolution of specific heat defined as *C*_V_ = (〈*E*^2^〉 − 〈*E*〉^2^)/*K*_B_*T*^2^ with temperature, from which we obtained the *T*_C_ of 290 K for CeF_2_ monolayer, by locating the peak position of *C*_V_. Since calculated *A* is much smaller than *J*, MAE of Ce ion is the minor reason for Curie temperature. The next-nearest neighboring (NNN) magnetic exchange parameter *J*_2_ can be calculated *via* the energy differences as shown in the (ESI†), when AFM-2 state is considered as shown in Fig. S3,† CeF_2_ monolayer is still FM, calculated *J*_2_ is 0.13 meV. Since NNN Ce–Ce bonds (6.33 Å) are much larger than that of NN Ce–Ce bonds (3.65 Å), both AFM and FM exchange interactions may be much weaker for NNN Ce ions, thus *J*_2_ is much smaller than *J*_1_ and can be neglected.

### Stability, electronic and magnetic properties of CeFCl monolayer

**Fig. 3:**
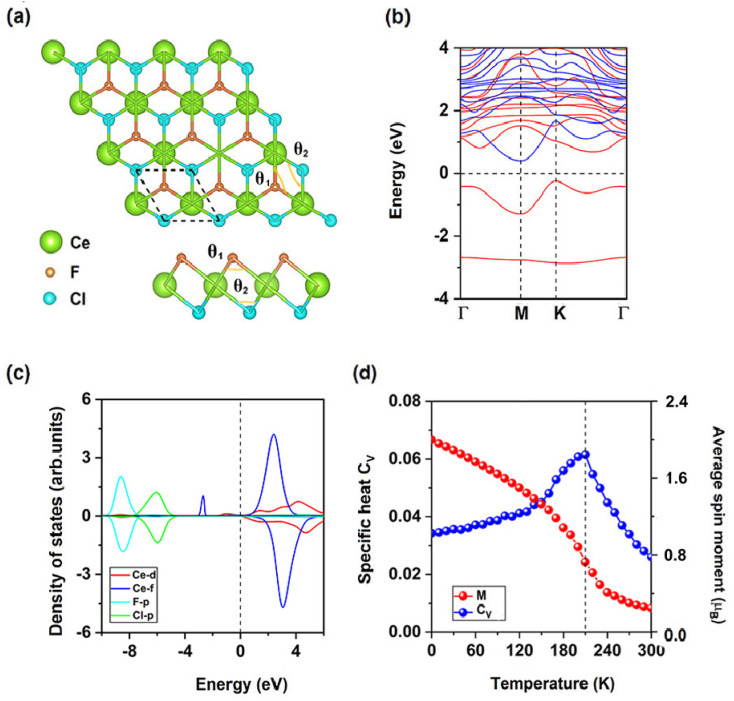
(a) Top and side views for the atomic structures, (b) spin-resolved electronic band structure, (c) projected density of states (PDOS) of Ce-d, Ce-f, F-p and Cl-p orbitals, (d) the simulated averaged net magnetic moment of CeFCl monolayer per formula unit and specific heat with respect to temperature for CeFCl monolayer.

By replacing one of the two F atomics layers to Cl atomic layer, CeFCl monolayer can be obtained, it has the same space group as the CeF_2_ monolayer. The ground state for CeFCl monolayer is FM state, the energy of the FM state is lower than that of AFM state by 65.15 meV per formula unit, the total magnetic moment for CeFCl monolayer is 2 *μ*_B_ per formula unit, magnetic moments for F ions, Cl ions, Ce ions and interstitial are −0.01 *μ*_B_, 0.01 *μ*_B_, 1.42 *μ*_B_ and 0.58 *μ*_B_, respectively. The optimized lattice constant of CeFCl monolayer is 3.77 Å, the nearest Ce–F and Ce–Cl bonds are 2.48 Å and 2.86 Å, respectively, since the angle *θ*_1_ (*θ*_2_) between nearest Ce ions and F(Cl) ion is 98.6° (82.7°) (Fig. 3(a)), thus super-exchange interactions between NN Ce ions *via* F or Cl ions can both induce FM.

**Fig. 4:**
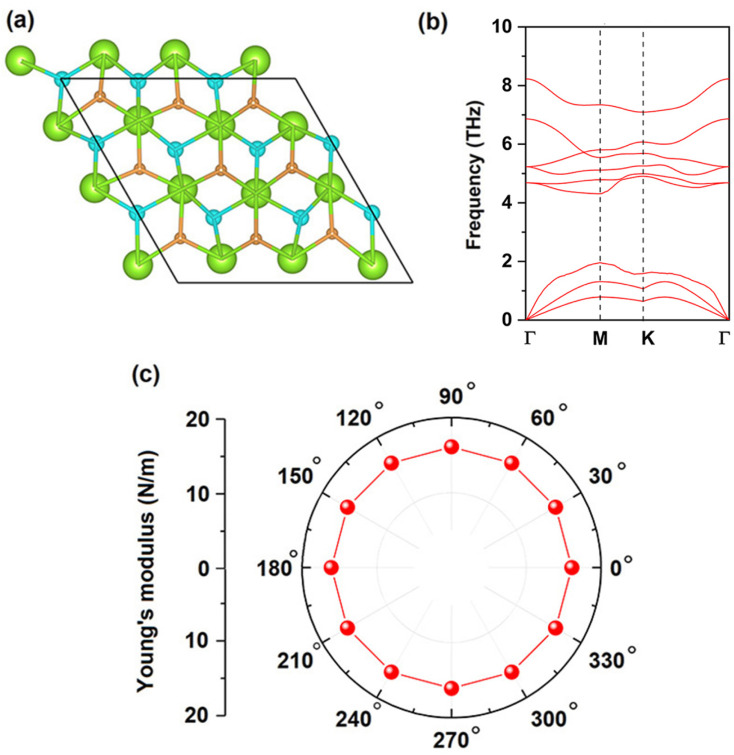
(a) Snapshots of atomic configurations at the end of 5 ps AIMD simulation at 300 K, (b) phonon spectrum, (c) angular dependence of the Young's modulus for CeFCl monolayer.

Different from CeF_2_ monolayer, CeFCl monolayer can maintain its intact structure at 300 K (Fig. 4(a)), the formation process is also exothermic because the formation energy is −8.79 eV per formula unit, the phonon spectrum confirms its dynamics stability as shown in Fig. 4(b), the calculated elastic constants *C*_11_, *C*_22_, *C*_12_ and *C*_66_ for CeFCl monolayer are 16.89 N m^−1^, 16.89 N m^−1^, 3.69 N m^−1^ and 6.60 N m^−1^, respectively, which satisfy with the Born–Huang criteria.^60^ The Young's modulus is isotropic due to the lattice symmetry as shown in Fig. 4(c), taking *L* ≈ 100 μm as an example, according to the equation mentioned above, the gravity induced deformation is also in the order of 10^−4^, thus can be neglected.

As shown in Fig. 3(b), CeFCl monolayer is a bipolar semiconductor with a band gap of 0.65 eV. The VBM (−0.25 eV) is attributed to spin-up channel while CBM (0.40 eV) is attributed to another channel, the vacuum level is 3.11 eV. According to the Fermi–Dirac distribution,^48^ the hopping possibility for electrons between VBM and conduction band in the spin-up channel (0.92 eV) is only in the order of 10^−13^ at 300 K, much less than that of CeF_2_ monolayer. Combined with its thermal instability, the CeFCl monolayer is more applicable than CeF_2_ monolayer. Both occupied F-p and Cl-p electrons are located deeply and Ce-f electrons are highly localized, so the occupied Ce-d electrons near the Fermi level are dominant to induce FM super-exchange interaction like CeF_2_ monolayer (Fig. 3(c)). Similar to CeF_2_ monolayer, CeFCl monolayer also has large MAE, that is −2.10 meV per formula unit, the MAE is mainly from Ce-f electrons (−2.25 meV). Calculated magnetic exchange parameter *J* for NN Ce ions and magnetic anisotropy parameter *A* are 4.07 meV and 0.53 meV, respectively, magnetic exchange parameter *J*_2_ for NNN Ce ions is 0.09 meV thus can be neglected, when AFM-2 state is considered, CeFCl monolayer is still FM, the net magnetic moment is 2 *μ*_B_ per formula unit and the obtained Curie temperature based on Heisenberg model is 210 K for CeFCl monolayer (Fig. 3(d)), higher than liquid-nitrogen temperature (77 K). There are following reasons why the Curie temperature for CeFCl monolayer is less than that of the CeF_2_ monolayer: (i), occupied Ce-d electrons near the Fermi level for CeFCl monolayer are less than those of the CeF_2_ monolayer; (ii), for CeFCl monolayer, interstitial magnetic moment is larger; (iii), both the angle *θ*_1_ and *θ*_2_ are more deviation from 90°, all these reasons weaken effective FM coupling between Ce ions, and result in lower Curie temperature.

### The effect of biaxial strain for CeFCl monolayer

**Fig. 5:**
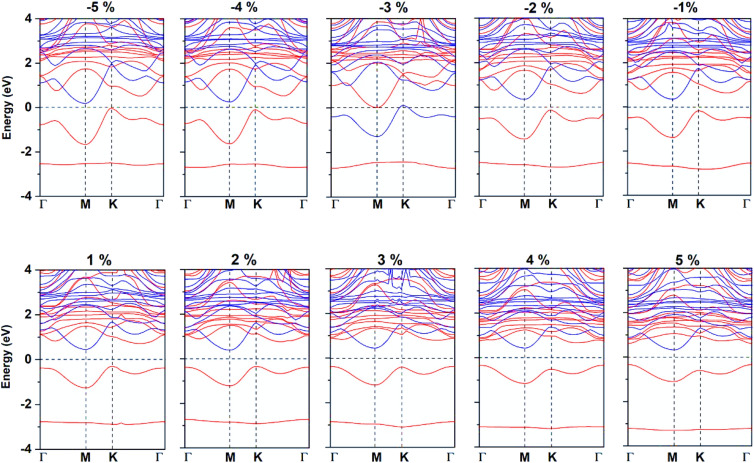
Spin-resolved band structure for CeFCl monolayer under biaxial strain from −5% to 5%.

Strain is an effective way to tune the electronic and magnetic properties, in this paper, we further studied the electronic and magnetic properties for CeFCl monolayer under biaxial strain ranging from −5% to 5%. We discussed FM and AFM states to verify its ground state, as shown in Fig. 5, calculation results show that the CeFCl monolayer is still FM. Moreover, the total magnetic moment is still 2 *μ*_B_ per formula unit and mainly attributed to Ce ion besides at −3% strain. At −3% strain, Ce ion is located in the low-spin state and magnetic moment is only 0.17 *μ*_B_, Ce ion is AFM coupling with both F (−0.02 *μ*_B_) and Cl ions (−0.05 *μ*_B_), thus total magnetic moment is only 0.05 *μ*_B_ per formula unit. Moreover, at −3% strain, both VBM and CBM touch the Fermi level slightly, thus the CeFCl monolayer turns to be metallic, while in other cases the CeFCl monolayer is still semiconducting, the VBM is attributed to the spin-up channel and the CBM is attributed to another channel. According to the crystal orbital Hamiltonian population (COHP) between Ce-d/f orbitals and F/Cl-p orbitals (Fig. S4†), the positive/negative values denote the bonding/anti-bonding states between these orbitals, thus near the Fermi level, interactions between Ce and F ions are stronger and contribute more to the FM exchange interactions. The interactions between Ce and F/Cl ions near the Fermi level become more and more weaker under biaxial strain from −5% to 4%, we conclude AFM direct interactions between Ce ions decreased more rapidly, thus Curie temperature increases in these cases. Moreover, at −3% strain, the anti-bonding states mainly between Ce-d and F-p orbitals cross the Fermi level thus the semiconducting characteristic for CeFCl monolayer is destroyed.

**Fig. 6:**
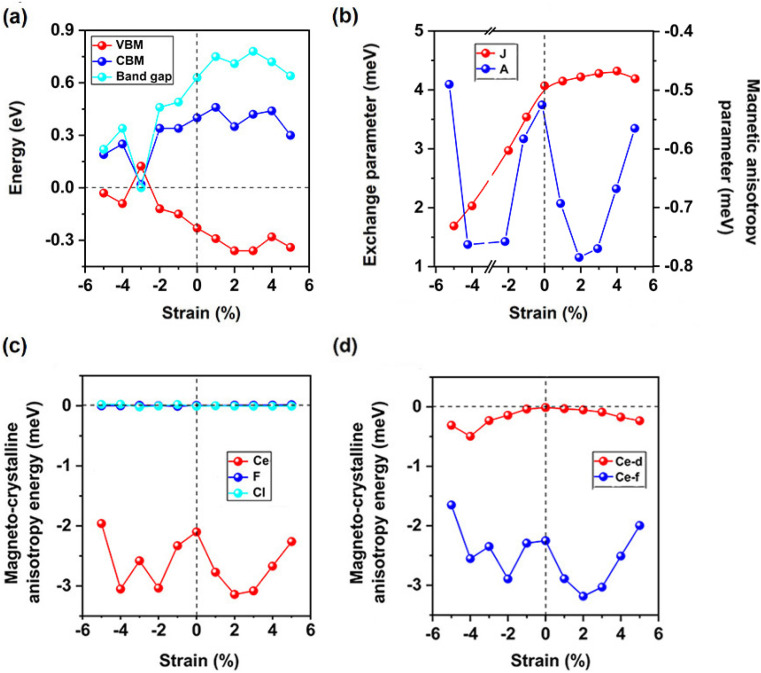
(a) Valence band maximum (VBM), conduction band minimum (CBM), and band gap in the semiconducting channel, (b) exchange parameter *J* and magnetic anisotropy parameter *A*, the magneto-crystalline anisotropy energies (MAE) of (c) Ce, F, and Cl ions, and projected on (d) Ce-d/f orbitals for CeFCl monolayer under biaxial strain from −5% to 5%.

Under compressive strain, the band gap, VBM and CBM are all decreased, while the band gap is increased under the strain smaller than 2%, then slightly decreased at 2% strain and reaches the maximum of 0.78 eV at 3% strain. Fig. 6(a) further indicates the increasing VBM for CeFCl monolayer under strain smaller than 2%, the VBM maintains the maximum of −0.36 eV at 3% strain, the CBM changes little under the tensile strain, it reaches the maximum of 0.46 eV at 1% strain. To further considered potential applications related to type-I^65^ and type-II^66^ composite heterojunctions based on semiconducting CeFCl monolayer, band alignments under biaxial strain are presented in Fig. S5,† the positions of VBM/CBM were obtained from the differences between the electrostatic potential at vacuum region. The positions of VBM range from −3.01 to −3.34 eV while the positions of CBM range from −2.27 to −3.11 eV, respectively, thus heterojunctions consist of CeFCl monolayer under biaxial strain from −2% to 5% and equilibrium ScBr_3_ and BiI_3_ (ref. 67) monolayers may be a series of promising type-I ones which can effectively inhibit leakage.

We choose net magnetic moment is 2 *μ*_B_ per formula unit besides at −3% strain. Fig. 6(b) shows that magnetic exchange parameters *J* increases from 1.69 meV to 4.31 meV under the strain up to 4%, but it slightly decreases to 4.19 meV at 5% strain, when AFM-2 state is considered, magnetic exchange parameter *J*_2_ is still close to 0, and CeFCl monolayer is still FM. Since magnetic anisotropy parameters *A* range from −0.49 meV to −0.79 meV (Fig. 6(b)), much smaller than *J*, magnetic anisotropy is still the minor factor for Curie temperature under biaxial strain.

**Fig. 7:**
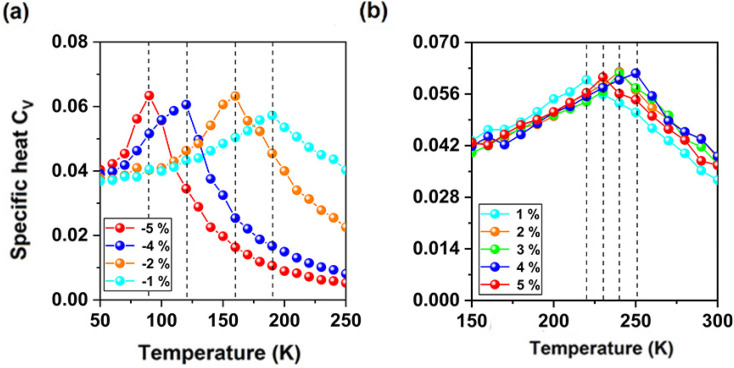
(a) and (b) the simulated averaged specific heat with respect to temperature for CeFCl monolayer under biaxial strain from −5% to 5%.

As shown in Fig. 6(c), under biaxial strain from −5% to 5%, the MAE of CeFCl monolayer is still large and the easy magnetization axes are along the out-of-plane directions, the MAE for F and Cl ions are close to zero, thus the MAE of Ce ion is nearly equal to the total MAE. Under tensile strain, total MAE becomes larger, at 2% strain, the MAE for Ce ion is the largest (−3.14 meV), under compressive strain from −1% ∼ −4%, total MAE is also larger and can increased to 3.05 meV, while it is slightly decreased to 1.96 meV at −5% strain. Comparing with Ce-f orbitals, the MAE for Ce-d orbitals can be neglected (Fig. 6(d)). As shown in Fig. 7(a) and (b), when considered spin Hamilton including both *J* and *A*, based on Heisenberg model, the Curie temperatures for CeFCl monolayer under compressive strain from −5% to −1% are 90 K, 120 K, 160 K, 190 K, respectively, while under tensile strain from 1% to 5%, the Curie temperatures for CeFCl monolayer are 220 K, 240 K, 240 K, 250 K, 230 K, respectively.

**Fig. 8:**
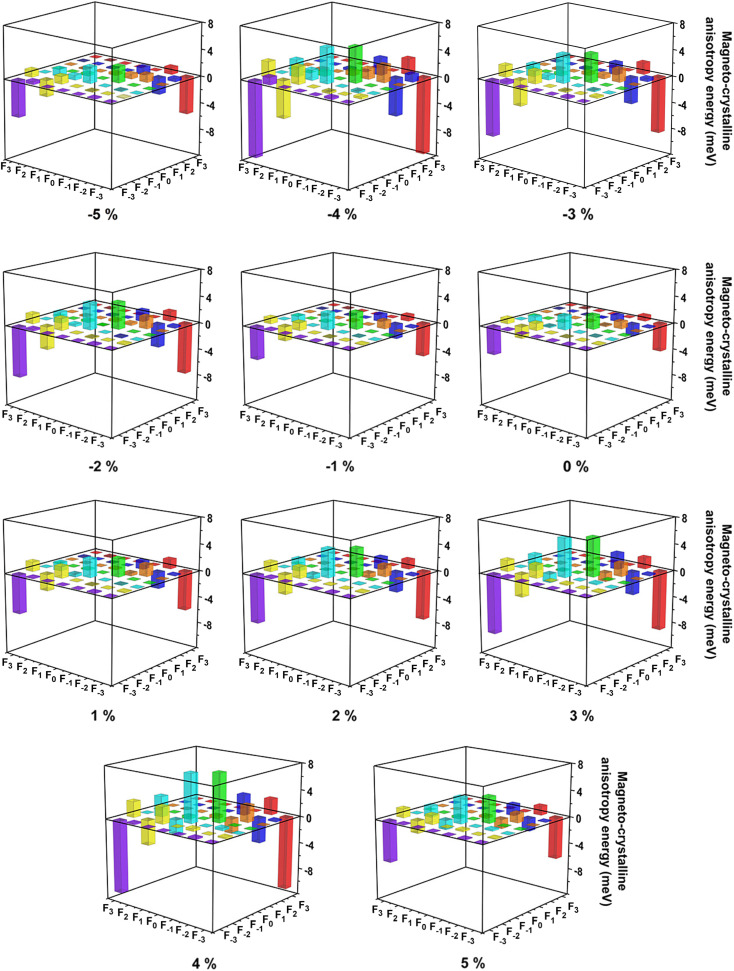
Resolved magneto-crystalline anisotropy energies (MAE) of Ce-f orbitals for CeFCl monolayer under biaxial strain from −5% to 5%, F_−3_, F_−2_, F_−1_, F_0_, F_1_, F_2_ and F_3_ orbitals represent f_*y*(3*x*^2^–*y*^2^)_^,^ f_*xyz*_, f_*yz*^2^_, f_*z*^3^_, f_*xz*^2^_, f_*z*(*x*^2^–*y*^2^)_ and f_*x*(*x*^2^–3*y*^2^)_ orbitals, respectively.

According to the Tables S1 and S2,† when two Ce f-orbitals in the same spin channel, two Ce f-orbitals coupling through in-plane (*x* or *y*) directions are all equal to 0, while through *z* direction, the diagonal non-zero matrix elements 〈*n*|*Ŝ*·*L̂*|*m〉* represent higher spin orbit coupling energies along *z* direction based on second-order perturbation theory.^68^ In a similar way, when two Ce f-orbitals in the different spin channels, non-zero matrix elements indicate corresponding directions are hard magnetization axes. As shown in Fig. 8, under biaxial strain from −5% to 5%, the couplings between Ce f_*y*(3*x*^2^–*y*^2^)_ and f_*x*(*x*^2^–3*y*^2^)_ orbitals are still dominant for magnetization along the out-of-plane directions, while the couplings between Ce f_*yz*^2^_ and f_*z*^3^_ orbitals are important for magnetization along the in-plane directions, since the coupling between Ce f_*y*(3*x*^2^–*y*^2^)_ and f_*x*(*x*^2^–3*y*^2^)_ orbitals are still stronger, thus the easy magnetization axes are along the out-of-plane directions under the biaxial strain from −5% to 5%. According to Table S2,† the couplings between Ce f_*y*(3*x*^2^–*y*^2^)_ and f_*x*(*x*^2^–3*y*^2^)_ orbitals in the different spin channels contribute most to magnetization along the out-of-plane directions.

### The discussion about experimental fabrication of CeFCl monolayer

According to the above calculation results, CeFCl monolayer is predicted to be a kind of novel FM semiconductor with great electronic and magnetic properties, however, there are not relevant experimental reports for CeFCl monolayer up to now, this phenomenon may attribute to less attentions about Ce-based compounds and the rarity of Ce element. To date, Jungmann and coworkers have successfully fabricated bulk CeI_2_ by syn-proportion of the triiodides with Ce-metal in arc-welded tantalum tubes,^69^ since both bulk CeF_3_ (ref. 70) and CeCl_3_ (ref. 71) had been synthesized several decades, we hope bulk CeF_2_ or CeCl_2_ can also be obtained in a similar way, then using micro-mechanical exfoliation method, monolayers CeF_2_ and CeCl_2_ can be fabricated and finally CeFCl monolayer can be obtained by the replacement of the F/Cl atoms by Cl/F atoms like MoSSe^72^ and WSSe^73^ monolayers. Moreover, Mihalyuk and coworkers have synthesized monolayers LnX_2_ (Ln = Gd, Eu; X = Si, Ge) by using the molecular-beam epitaxial method,^74^ since the structure of CeFCl monolayer is similar to that of monolayers LnX_2_ (Ln = Gd, Eu; X = Si, Ge), we also hope CeFCl monolayer can be synthesized in this way.

## Conclusion

In summary, the stability, electronic and magnetic properties of CeF_2_ and CeFCl monolayers have been carefully investigated. Our results show that CeF_2_ monolayer is ferromagnetic (FM) semiconductor with 290 K Curie temperature, 0.40 eV band gap, and sizable magneto-crystalline anisotropy energy (MAE) of −2.90 meV per formula unit. Its total magnetic moment is 2 *μ*_B_ per formula unit and mainly comes from the Ce ion, plus, its mechanical and dynamical stability have been confirmed, both larger possibility for electronic hopping and thermal instability indicate CeF_2_ monolayer is not suitable at higher temperature. The CeFCl monolayer is a FM bipolar semiconductor with 210 K Curie temperature, 0.65 eV band gap and −2.10 meV MAE per formula unit, its stable semiconductor properties indicate it is more suitable for practical applications. Under biaxial strain from −5% to 5%, its semiconducting characteristic can be well preserved besides at −3% strain, tensile strain can enhance its Curie temperature up to 250 K, the couplings between Ce f_*y*(3*x*^2^–*y*^2^)_ and f_*x*(*x*^2^–3*y*^2^)_ orbitals in the different spin channels are dominant for the easy magnetization axes, the easy magnetization axes are along the out-of-plane directions under biaxial strain from −5% to 5%.

## Data availability

The data supporting of this paper have been included as part of the ESI.†

## Conflicts of interest

There are no conflicts to declare.

## Supplementary Material

RA-015-D4RA06728B-s001
