# Supplementary material for: First-principles predictions of two-dimensional Ce-based ferromagnetic semiconductors: CeF2 and CeFCl monolayers
Source: RSC Adv. 2025 Jan 23;15(3):2163–74. doi: 10.1039/d4ra06728b (PMC11755109; doi:10.1039/d4ra06728b)
Supplement: RA-015-D4RA06728B-s001 [file RA-015-D4RA06728B-s001.pdf]

This journal is © The Royal Society of Chemistry 2025

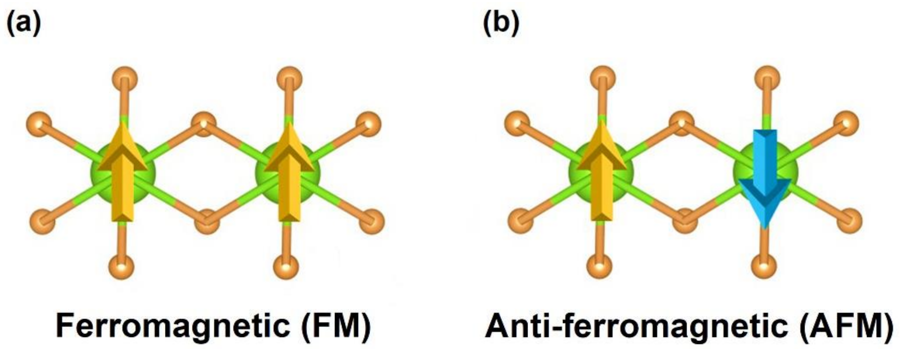

Fig. S1. Schematic diagrams for (a) ferromagnetic and (b) anti-ferromagnetic configurations for CeF_2 and CeFCl monolayers.

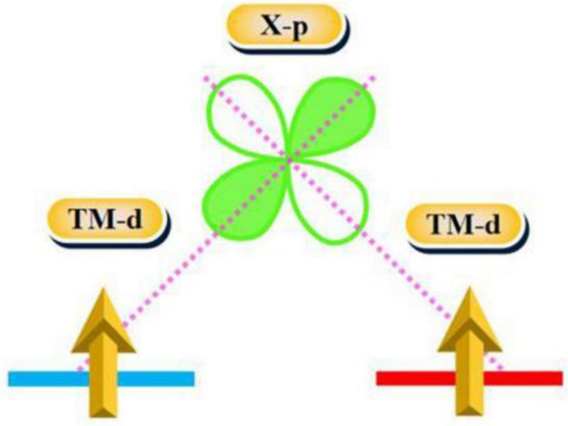

Fig. S2. The ferromagnetic super-exchange interaction according to the Goodenough-Kanamori-Anderson (GKA) rule.

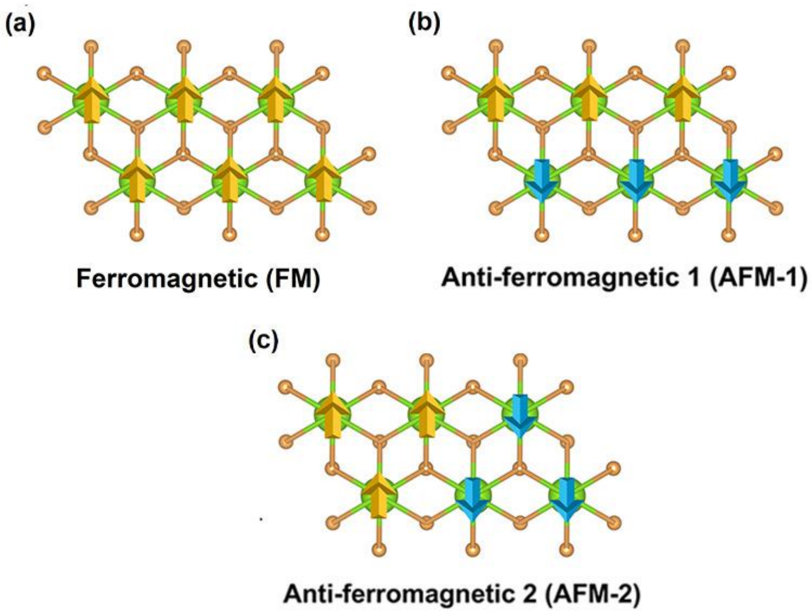

Fig. S3. Schematic diagrams for (a) ferromagnetic, (b) anti-ferromagnetic 1 (AFM-1), and (c) anti-ferromagnetic 2 (AFM-2) configurations for CeF_2 and CeFCl monolayers.

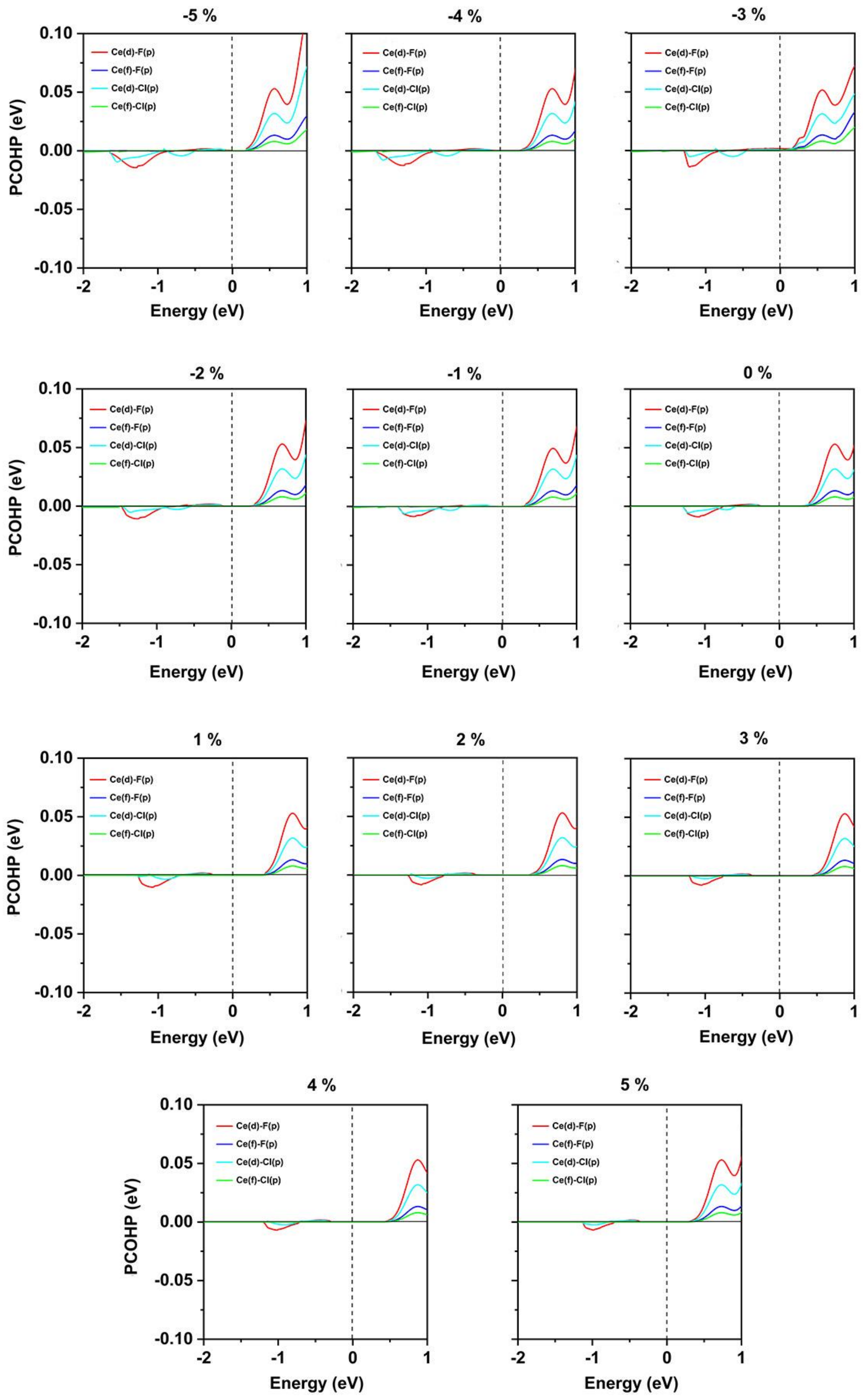

Fig. S4. The orbital-projected crystal orbital Hamilton population (PCOHP) for CeFCl monolayer under biaxial strain from -5 % to 5 %.

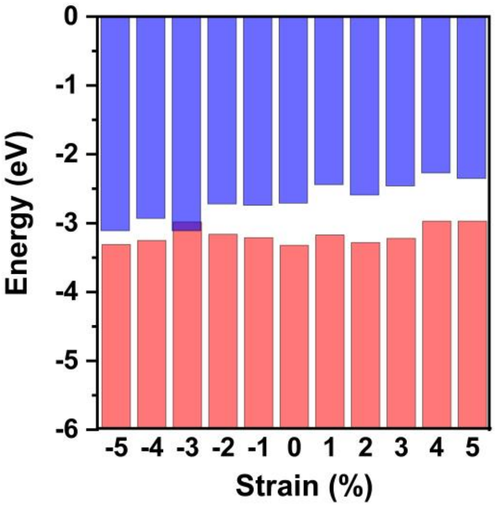

Fig. S5. Band alignments of CeFCl monolayer under biaxial strain from -5 % to 5 %, the vacuum level is set as 0 eV.

Calculation details for next-nearest magnetic exchange parameters J_2

For both CeF_2 and CeFCl monolayers, each Ce ion has 3 nearest Ce ions and 3 next-nearest Ce ions, the spin-Hamiltonian including nearest neighboring (NN) and next-nearest neighboring (NNN) magnetic exchange parameters J_1 and J_2 is described as:

$$H = -\sum_{i,j} J_1 M_i M_j - \sum_{k,l} J_2 M_k M_l \tag{1}$$

where J_1 and J_2 are NN and NNN magnetic exchange parameter respectively, M is the net magnetic moment of Ce ions, (i, j) and (k, l) stand for the NN and NNN pairs of Ce ions. For FM, AFM-1, and AFM-2 states as shown in Figs. S3, in the $3\times2\times1$ supercell, the spin-Hamiltonian can be written as:

$$E_{FM} = -18J_1M^2 - 18J_2M^2 \tag{2}$$

$$E_{AFM-1} = 6J_1M^2 + 6J_2M^2 \tag{3}$$

$$E_{AFM-2} = 2J_1M^2 + 6J_2M^2 \tag{4}$$

Both J_1 and J_2 are calculated via the energy differences between the FM and AFM-1 (AFM-2) states in the $3\times2\times1$ supercell as:

$$J_1 = \frac{E_{AFM-1} - E_{AFM-2}}{4M^2} \tag{5}$$

$$J_2 = \frac{E_{AFM-1} - E_{FM}}{24M^2} - J_1 \quad (6)$$

The second-order perturbation theory including spin-orbital coupling (SOC)

According to the second-order perturbation theory, interactions from spin-orbit coupling (SOC) can be regard as perturbation, thus the Hamiltonian including SOC is,

$$H = H(0) + \lambda^2 \sum_{m \neq n} \frac{|< n|\hat{S} \cdot \hat{L}|m>|^2}{E_n^{(0)} - E_m^{(0)}} \quad (7)$$

where λ is the SOC constant, $\hat{S}$ and $\hat{L}$ are the spin momentum operator and orbital momentum operator, respectively, $|n>$ and $|m>$ represent the eigenstates of the Hamiltonian quantum states, $E_n^{(0)}$ and $E_m^{(0)}$ denote their eigenvalues, respectively. When spin-up ($|\uparrow>$) and spin-down ($|\downarrow>$) states are considered, the corresponding energy from the SOC interaction[1] is:

$$E^{SOC} = \lambda^2 \Big(\sum_{m \neq n} \frac{|<\uparrow n|\hat{S} \cdot \hat{L}|\uparrow m>|^2}{E_n^{(0)} - E_m^{(0)}} + \sum_{m \neq n} \frac{|<\uparrow n|\hat{S} \cdot \hat{L}|\downarrow m>|^2}{E_n^{(0)} - E_m^{(0)}} + \sum_{m \neq n} \frac{|<\downarrow n|\hat{S} \cdot \hat{L}|\uparrow m>|^2}{E_n^{(0)} - E_m^{(0)}} + \sum_{m \neq n} \frac{|<\downarrow n|\hat{S} \cdot \hat{L}|\downarrow m>|^2}{E_n^{(0)} - E_m^{(0)}} \Big) \quad (8)$$

For $|<\uparrow n|\hat{S} \cdot \hat{L}|\uparrow m>|^2$ and $|<\downarrow n|\hat{S} \cdot \hat{L}|\downarrow m>|^2$, the $\hat{S} \cdot \hat{L}$ term can be written as $\hat{Sz} \cdot \hat{Lz}$, the eigenvalue of $\hat{Sz}$ is still $\frac{1}{2}$, while the eigenvalue of $\hat{Lz}$ depends on magnetic quantum number for $|m>$ quantum states[2]:

$$\hat{Lz}\, Y_l^m = m Y_l^m \quad (9)$$

For $|<\uparrow n|\hat{S} \cdot \hat{L}|\downarrow m>|^2$ and $|<\downarrow n|\hat{S} \cdot \hat{L}|\uparrow m>|^2$, the $\hat{S} \cdot \hat{L}$ term is different from mentioned before and can be written as $\frac{1}{2}(\hat{S}_+ \cdot \hat{L}_- + \hat{S}_- \cdot \hat{L}_+)$, the $\hat{S}_+$, $\hat{S}_-$, $\hat{L}_+$ and $\hat{L}_-$ are ladder operators which can be denotes as[2]:

$$\hat{S}_+ = \hat{S}_x + i\hat{S}_y\,, \quad \hat{S}_- = \hat{S}_x - i\hat{S}_y \quad (10)$$

$$\hat{L}_+ = \hat{L}_x + i\hat{L}_y\,, \quad \hat{L}_- = \hat{L}_x - i\hat{L}_y \quad (11)$$

their eigenvalues can be expressed by the following formula[2]:

$$\hat{S}_{+}|\uparrow>=0,\ \hat{S}_{+}|\downarrow>=|\uparrow>,\ \hat{S}_{-}|\uparrow>=|\downarrow>,\ \hat{S}_{-}|\downarrow>=0 \tag{12}$$

$$\hat{L}_{\pm}Y_l^m=\sqrt{(l\mp m)(l\pm m+1)}Y_l^{m\pm1} \tag{13}$$

Since all the Ce-4f orbits can be expressed by spherical harmonic function as shown in Table. S1[3], taking equations (9-13) into the equation (8), we can get matrix elements $<n|\hat{S}\cdot\hat{L}|m>$ as shown in the following Tables S2.

Table. S1. The angular quantum number, magnetic quantum number, and corresponding orbits expressed by spherical harmonic function.

l	m	Orbits
	-3	$4f_{y(3x^2-y^2)}=i/\sqrt{2}\ [Y_3^{-3}(\theta,\varphi)+Y_3^3(\theta,\varphi)]$
	-2	$4f_{xyz}=i/\sqrt{2}\ [Y_3^{-2}(\theta,\varphi)-Y_3^2(\theta,\varphi)]$
	-1	$4f_{yz^2}=i/\sqrt{2}\ [Y_3^{-1}(\theta,\varphi)+Y_3^1(\theta,\varphi)]$
3	0	$4f_{z^3}=\ Y_3^0(\theta,\varphi)$
	1	$4f_{xz^2}=1/\sqrt{2}\ [Y_3^{-1}(\theta,\varphi)-Y_3^1(\theta,\varphi)]$
	2	$4f_{z(x^2-y^2)}=1/\sqrt{2}\ [Y_3^{-2}(\theta,\varphi)+Y_3^2(\theta,\varphi)]$
	3	$4f_{x(x^2-3y^2)}=1/\sqrt{2}\ [Y_3^{-3}(\theta,\varphi)-Y_3^3(\theta,\varphi)]$

Table. S2. Matrix elements $<n|\hat{S}\cdot\hat{L}|m>$ of the spin-orbit coupling operator, the directional unit vector $\vec{n}$ represent the direction of the spin state, $|n| = \sqrt{x^2 + y^2 + z^2}$, where x, y and z are directional cosines.

	$\|\uparrow, f_{y(3x^2-y^2)}>$	$\|\uparrow, f_{xyz}>$	$\|\uparrow, f_{yz^2}>$	$\|\uparrow, f_{z^3}>$	$\|\uparrow, f_{xz^2}>$	$\|\uparrow, f_{z(x^2-y^2)}>$	$\|\uparrow, f_{x(x^2-3y^2)}>$
$\|\uparrow, f_{y(3x^2-y^2)}>$	0	0	0	0	0	0	1.5i (3iz)
$\|\uparrow, f_{xyz}>$	0	0	0	0	0	i (2iz)	0
$\|\uparrow, f_{yz^2}>$	0	0	0	0	0.5i (iz)	0	0
$\|\uparrow, f_{z^3}>$	0	0	0	0	0	0	0
$\|\uparrow, f_{xz^2}>$	0	0	-0.5i (-iz)	0	0	0	0
$\|\uparrow, f_{z(x^2-y^2)}>$	0	-i (-2iz)	0	0	0	0	0
$\|\uparrow, f_{x(x^2-3y^2)}>$	-1.5i (-3iz)	0	0	0	0	0	0

	$\|\downarrow, f_{y(3x^2-y^2)}>$	$\|\downarrow, f_{xyz}>$	$\|\downarrow, f_{yz^2}>$	$\|\downarrow, f_{z^3}>$	$\|\downarrow, f_{xz^2}>$	$\|\downarrow, f_{z(x^2-y^2)}>$	$\|\downarrow, f_{x(x^2-3y^2)}>$
$\|\downarrow, f_{y(3x^2-y^2)}>$	0	0	0	0	0	0	1.5i (-3iz)
$\|\downarrow, f_{xyz}>$	0	0	0	0	0	i (-2iz)	0
$\|\downarrow, f_{yz^2}>$	0	0	0	0	0.5i (-iz)	0	0
$\|\downarrow, f_{z^3}>$	0	0	0	0	0	0	0
$\|\downarrow, f_{xz^2}>$	0	0	-0.5i (iz)	0	0	0	0
$\|\downarrow, f_{z(x^2-y^2)}>$	0	-i (2iz)	0	0	0	0	0
$\|\downarrow, f_{x(x^2-3y^2)}>$	-1.5i (3iz)	0	0	0	0	0	0

	$\|\downarrow, f_{y(3x^2-y^2)}>$	$\|\downarrow, f_{xyz}>$	$\|\downarrow, f_{yz^2}>$	$\|\downarrow, f_{z^3}>$	$\|\downarrow, f_{xz^2}>$	$\|\downarrow, f_{z(x^2-y^2)}>$	$\|\downarrow, f_{x(x^2-3y^2)}>$
$\|\uparrow, f_{y(3x^2-y^2)}>$	0	$\sqrt{6}\, iy/2$	0	0	0	$\sqrt{6}\, ix/2$	0
$\|\uparrow, f_{xyz}>$	$-\sqrt{6}\, iy/2$	0	$\sqrt{10}\, y/2$	0	$\sqrt{10}\, ix/2$	0	$\sqrt{6}\, ix/2$
$\|\uparrow, f_{yz^2}>$	0	$-\sqrt{10}\, y/2$	0	$-\sqrt{6}\, ix$	0	$-\sqrt{10}\, ix/2$	0
$\|\uparrow, f_{z^3}>$	0	0	$\sqrt{6}\, ix$	0	$\sqrt{6}\, iy$	0	0
$\|\uparrow, f_{xz^2}>$	0	$-\sqrt{10}\, ix/2$	0	$-\sqrt{6}\, iy$	0	$\sqrt{10}\, iy/2$	0
$\|\uparrow, f_{z(x^2-y^2)}>$	$-\sqrt{6}\, ix/2$	0	$\sqrt{10}\, ix/2$	0	$-\sqrt{10}\, iy/2$	0	$\sqrt{6}\, iy/2$
$\|\uparrow, f_{x(x^2-3y^2)}>$	0	$-\sqrt{6}\, ix/2$	0	0	0	$-\sqrt{6}\, iy/2$	0

	$\|\uparrow, f_{y(3x^2-y^2)}>$	$\|\uparrow, f_{xyz}>$	$\|\uparrow, f_{yz^2}>$	$\|\uparrow, f_{z^3}>$	$\|\uparrow, f_{xz^2}>$	$\|\uparrow, f_{z(x^2-y^2)}>$	$\|\uparrow, f_{x(x^2-3y^2)}>$
$\|\downarrow, f_{y(3x^2-y^2)}>$	0	$-\sqrt{6}\, iy/2$	0	0	0	$\sqrt{6}\, ix/2$	0
$\|\downarrow, f_{xyz}>$	$\sqrt{6}\, iy/2$	0	$-\sqrt{10}\, y/2$	0	$-\sqrt{10}\, ix/2$	0	$-\sqrt{6}\, ix/2$
$\|\downarrow, f_{yz^2}>$	0	$\sqrt{10}\, y/2$	0	$\sqrt{6}\, ix$	0	$\sqrt{10}\, ix/2$	0
$\|\downarrow, f_{z^3}>$	0	0	$-\sqrt{6}\, ix$	0	$-\sqrt{6}\, iy$	0	0
$\|\downarrow, f_{xz^2}>$	0	$\sqrt{10}\, ix/2$	0	$\sqrt{6}\, iy$	0	$-\sqrt{10}\, iy/2$	0
$\|\downarrow, f_{z(x^2-y^2)}>$	$\sqrt{6}\, ix/2$	0	$-\sqrt{10}\, ix/2$	0	$\sqrt{10}\, iy/2$	0	$-\sqrt{6}\, iy/2$
$\|\downarrow, f_{x(x^2-3y^2)}>$	0	$\sqrt{6}\, ix/2$	0	0	0	$\sqrt{6}\, iy/2$	0

References

1. D. S. Wang, R. Wu, and A. J. Freeman, Phys Rev B, 1993, 47, 14932.
2. W. A. Harrison, Applied quantum mechanics, World Publishing Corporation, 2003.
3. C. D. H. Chisholm. Group theoretical techniques in quantum chemistry. Academic Press, 1976.
